# Genetic Association of SNPs near *ATOH7*, *CARD10*, *CDKN2B*, *CDC7* and *SIX1/SIX6* with the Endophenotypes of Primary Open Angle Glaucoma in Indian Population

**DOI:** 10.1371/journal.pone.0119703

**Published:** 2015-03-23

**Authors:** Ferdinamarie Sharmila Philomenadin, Rashima Asokan, Viswanathan N, Ronnie George, Vijaya Lingam, Sripriya Sarangapani

**Affiliations:** 1 SNONGC Department of Genetics and Molecular biology, Vision Research foundation, Sankara Nethralaya, Chennai, India; 2 PhD Scholar, Birla Institute of Technology & Science (BITS), Pilani, 333 031, Rajasthan, India; 3 Chennai Glaucoma Study, Medical and Vision Research Foundation, Sankara Nethralaya, Chennai, India; 4 Biostatistician, Department of Preventive Ophthalmology, Medical research foundation, Sankara Nethralaya, Chennai, India; Casey Eye Institute, UNITED STATES

## Abstract

Primary open angle glaucoma (POAG) belonging to a group of optic neuropathies, result from interaction between genetic and environmental factors. Study of associations with quantitative traits (QTs) is one of the successful strategies to understand the complex genetics of POAG. The current study attempts to explore the association of variations near/in genes like *ATOH7*, *SIX1/SIX6* complex, *CDKN2B*, *CARD10*, and *CDC7* with POAG and its QTs including vertical cup to disc ratio (VCDR), central corneal thickness (CCT), intra ocular pressure (IOP), and axial length (AL). Case-control study design was carried out in a sample size of 97 POAG cases and 371 controls from South India. Model-based (additive, recessive, dominant) association of the genotypes and their interaction was carried out between cases and controls using chi-square, linear and logistic regression methods. Nominal significance (*P*<0.05) was observed for QTs like i) VCDR with SNPs rs1900004 (*ATOH7*); rs1192415 (*CDC7)*; rs10483727 (*SIX1/SIX6)*, rs9607469 (*CARD10);* ii) CCT with rs1192415; iii) IOP with rs1900004 and iv) AL with rs1900004 and rs1063192 (*CDKN2B)*. We were able to replicate previously known interactions between *ATOH7-SIX6* and *SIX6-CDKN2B* along with few novel interactions between *ATOH7*—*CDC7* and *SIX6* with genes including *CARD10* and *CDC7*. In summary, our results suggest that a probable interaction among the candidate genes for QTs, play a major role in determining the individual’s susceptibility to POAG.

## Introduction

Glaucoma is a heterogeneous group of optic neuropathies characterized by changes in the optic nerve head and corresponding loss of visual field. It is the second largest cause of blindness worldwide, next to cataract. The disease affects about 67 million people worldwide and in India, POAG accounts for 1.5–11.1% of the total blindness [1,2,3,4,5]. Some of the major risk factors of POAG include increased intraocular pressure (IOP), age, ethnicity and family history [6]. The disease follows a complex inheritance pattern with the involvement of both genetics and environmental factors [7]. It has been estimated that the heritability of POAG is about 0.81[8] and the risk of disease development is 22% for the relative of an affected individual when compared with that of an unaffected person (2–3%) [9].

Till date 33 chromosomal loci have been linked with the disease of which 3 genes Myocilin (*MYOC*) [10], optineurin (*OPTN*) [11], and *WDR36* [12] have been analysed and shown to contribute to 5% of the disorder [13]. Genome wide association studies (GWAS) have identified several candidate genes to be significantly associated with POAG that includes *CAV1* and *CAV2* [14], *SRBD1* [15], *TMCO1*[16], *CDKN2B-AS1* [17]. However the genetics of POAG could not be completely deciphered as the disease by itself expresses as an additive effect of various clinical features like variation in IOP, difference in the field of vision loss, optic nerve head parameters; and thus several genes each with small effect contributes to the complete phenotype [9]. Hence the focus of gene mapping strategies for POAG has shifted towards mapping measurable traits (quantitative traits (QTs)) which offers the advantage of reducing possible genetic and phenotypic complexity of the disease. These traits, known as endophenotypes are highly heritable, exhibit genetic correlation and are associated with the disease. The endophenotypes of POAG include IOP, central corneal thickness (CCT), optic nerve head parameters (vertical cup to disc ratio (VCDR), optic disc area (ODA)) with heritable estimates of 0.94, 0.68, [18] and 0.48–0.8 [19] respectively. These endophenotypes have been associated with different genomic regions by GWAS and some of them have been replicated across different populations. Three polymorphisms rs1900004 (within 10kb of *ATOH7*) [20,21], rs10483727 (near *SIX1-SIX6*) and rs1063192 (in 3’UTR region of *CDKN2B*) [22] have been strongly associated with optic disc parameters such as ODA, optic cup area and VCDR in various populations [23]. The other genes include *CDC7*/*TGFBR3* (rs1192415) and *CARD10* (rs9607469) which have been associated with ODA in Asian cohort [21]. CCT has been associated with genes like *ZNF469* [24], *COL5A*, *COL8A2* [25,26], *FOXO1*, *FNDC3B* [24] and *AKAP13* [27] in both Caucasian and Asian cohorts. Genes associated with IOP include *TMCO1*, *CDKN2B-AS1*, *GAS7*, *CAV1/CAV2*, and *SIX1/SIX6* [28].

Till date, genetic analysis of POAG in Indian population were reported on Myocilin [29,30], *CYP1B1*, *OPTN* [31,32] and *NTF4* [33] genes; which does not explain completely the genetic aetiology of POAG. Recent report from East India, on evaluation of INK4 locus (*CDKN2B)* showed negative association with POAG [34]. All these justify the need for glaucoma QT association study in Indian population. The current study focuses on the association of endophenotypes of POAG with SNPs rs1900004 and rs3858145 (*ATOH7*), rs10483727 (*SIX1/SIX6*), rs1192415 (*CDC7*), rs1063192 (*CDNK2B*) and rs9607469 (*CARD10*) in South Indian cohort.

## Materials and Methods

### Sample size

The study was approved by the institutional ethics board, as per the guidelines in the Declaration of Helsinki and was conducted at the SNONGC Department of Genetics and Molecular Biology, Vision Research Foundation, Sankara Nethralaya, India. Ninety seven patients diagnosed with POAG (both high tension (HTG = 66) and normal tension glaucoma (NTG = 31)) were recruited from the glaucoma clinic of Sankara Nethralaya eye hospital and the Chennai glaucoma study, a population-based cross-sectional study on the prevalence of glaucoma in south India (Project no: 11–2003-P). A total of 7774 subjects aged 40 years and above were examined at a dedicated facility in the base hospital. The study had a response rate of 81% [35]. The selection criteria and methodology have been explained previously [36]. All subjects were included after obtaining written informed consent.

All subjects >40 years of age were enrolled and categorized as patients and controls after a detailed clinical examination. The comprehensive ophthalmic examination included measurement of best corrected visual acuity, slit lamp biomicroscopy, applanation tonometry, gonioscopy, pachymetry, dilated fundus examination that included stereobiomicroscopic evaluation of the optic disc and macula with a 78 D lens, and examination of the retina using the indirect ophthalmoscope, Humphrey visual fields, and optic disc documentation of patients >40 years of age were included in the study. Standardized inclusion criteria for NTG were used, which was the presence of glaucomatous optic neuropathy (defined as loss of neuroretinal rim with a cup:disc ratio of 0.6 or greater) with compatible visual field loss and open angles on gonioscopy, and a mean IOP without treatment that was consistently <21 mmHg. The control samples were age matched and selected after complete ophthalmic examination similar to that of the glaucoma cases.

### SNP Genotyping

Genomic DNA was extracted by Qiagen kit / Phenol chloroform method, from 10ml of heparinised blood sample. SNPs rs1900004 and rs3858145 (about 10kb at the 5’ upstream of *ATOH7* on 10q21.3–22.1), rs10483727 (within 40kb of *SIX1/SIX6* gene complex on 14q22–23), rs1192415 (within 117kb of CDC7on 1p22), rs1063192 (within *CDNK2B* on 9p21), and rs9607469 (at the 5’ upstream of *CARD10* on 22q13.1) were included in the present study. Genotyping of SNPs were done by i) PCR based restriction fragment length polymorphism method for SNPs rs1900004 (*NlaIII*), rs3858145 (*MfeI*), rs10483727(*NdeI*), and rs9607469 (*HhaI*) (ii) Allele specific PCR for rs1063192 as described by Mabuchi et al [23] and (iii) direct sequencing for rs1192415 in ABI Prism Avant 3100 genetic analyzer.

### Statistical analysis

Analyses of allele/ genotype frequencies for association were performed by χ^2^ test. All SNPs were analyzed for deviation from Hardy-Weinberg equilibrium (HWE). All the QTs were checked for normal distribution. The CCT values were normally distributed while the other QTs were skewed. We performed logarithmic and square root transformation for IOP (after removing the outliers) and VCDR respectively. The AL values resumed the normal distribution after the removing the outlier values.

Statistical analysis for testing the association of CCT, VCDR, IOP and AL with the SNPs was performed using linear regression analysis in SPSS software v17. The effect size (β ± SE; conveys the magnitude of the relationship of the SNPs with the endophenotypes in the study) was calculated for the 3 models with the codes 0,1,2 / 0,1,1 / 0,0,1 representing homozygous major, heterozygous and homozygous minor alleles for the additive/dominant/ recessive models respectively. Multiple comparisons were corrected using the Bonferroni method. Logistic regression analysis was used to analyse the gene-gene interaction among HTG and NTG with 2 models (dominant and recessive) after adjustment for age and gender; wherein the coded SNPs were used as explanatory variables.

## Results

A total of 97 POAG patients (NTG = 31; HTG = 66) and 371 controls were included in the current study. Table 1 describes the demographic and clinical features of the study subjects. All the endophenotypes were significantly distributed between the cases and controls (*P* value = <0.0001).

**Table 1:** Demographic and clinical features of the study subjects.

			age (y)*		IOP (mmHg)	VCDR	CCT (μm)	AL (mm)	ACD (mm)
	n	male	range	mean±SD	mean±SD	mean±SD	mean±SD	mean±SD	mean±SD
**Control**	371	161	42–84	59.1 ± 8.7	14.5 ± 3.2	0.4 ± 0.2	511.5 ± 31.7	22.59 ± 0.8	2.8 ± 0.3
**HTG**	66	45	45–89	64.42 ± 10.36	28.3 ± 6.5	0.7 ± 0.2	528.9 ± 30.9	23.9 ± 1.8	2.6 ± 0.8
**NTG**	31	24	44–74	59.39 ± 8	17.87± 2.7	0.77 ± 0.2	518.87 ± 30.1	24.04 ± 1.9	2.9 ± 0.5

*the age at recruitment for controls or age of disease onset for POAG patients are shown

SD-standard deviation, IOP- intraocular pressure, VCDR-vertical cup to disc ratio, CCT- central corneal thickness, AL- axial length, ACD- anterior chamber depth.

### Analysis for association of the SNPs with POAG

All the six SNPs genotyped in the present study did not show any deviation from HWE (all *P* >0.001). The distribution of genotypes (additive/dominant/recessive) and minor allele frequencies of all the 6 SNPs did not show any significant difference (*P>*0.05) between the cases and controls (χ^2^ analysis) (Table 2).

**Table 2:** Analysis of minor allele frequencies and χ^**2**^ of the SNPs between POAG and controls.

SNP	Near gene	M/m	Additive model						Dominant model	Recessive model
			Controls		Cases		GenotypicP*	Allelic P*	*P* *	*P* *
			N	(%)	N	(%)				
**rs1900004**	*ATOH7*	C/T	225	30.32	62	31.96	0.9	0.75	0.73	0.84
**rs3858145**	*ATOH7*	A/G	303	40.84	88	45.36	0.52	0.46	0.39	0.37
**rs10483727**	*SIX6*	T/C	304	40.97	82	42.27	0.14	0.11	0.06	0.244
**rs1063192**	*CDKN2B*	T/C	171	23.05	47	24.23	0.87	0.95	0.9	0.65
**rs9607469**	*CARD10*	G/A	217	29.25	52	26.8	0.78	0.67	0.49	0.84
**rs1192415**	*CDC7*	A/G	233	31.4	74	38.14	0.16	0.25	0.2	0.09

M- Major allele, m-minor allele

* *P* values are derived from χ^2^ tests; significance: P<0.05

### Analysis of association of SNPs with IOP, AL, CCT and VCDR in the whole cohort

Analysis of association of SNPs with IOP, AL, CCT, and VCDR were performed after adjustment for age and gender; IOP was adjusted as an additional covariate for CCT and vice versa. The Bonferroni corrected *P* value was set to < = 0.008 (0.05/6) (Table 3). SNP i) rs1192415 (*CDC7)* was associated with decreased CCT (*P* = 0.021 (β±SE = -10.9±4.7); recessive model) and increased VCDR (*P* = 0.027 (β±SE = 0.05±0.02); recessive model), ii) rs1900004 (*ATOH7)* was associated with decreased VCDR (*P* = 0.02 (β±SE = -0.03±0.01); additive, *P* = 0.029 (β±SE = -0.09±0.04); recessive model), and increased AL (*P* = 0.028 (β±SE = -0.83±0.4)). However none of these *P* values survived Bonferroni correction.

**Table 3:** Multiple linear regression analysis for the SNPs with AL, CCT, VCDR and IOP as dependant variable in the whole cohort.

SNP	Minor allele	Additive model				Dominant model				Recessive model			
		AL (n = 109)	CCT (n = 454)	VCDR (n = 426)	IOP (n = 448)	AL	CCT	VCDR	IOP	AL	CCT	VCDR	IOP
		P*	P*	P*	P*	P*	P*	P*	P*	P*	P*	P*	P*
**rs1900004**	T	0.07	0.74	**0.02** ^Ω^ **(−0.03 ±0.01**)	0.19	0.34	0.97	0.1	0.17	**0.028** ^Ω^ **(0.83±0.4**)	0.59	**0.029** ^Ω^ **(−0.09 ±0.04**)	0.59
**rs3858145**	G	0.38	0.19	0.06	0.88	0.13	0.43	0.22	0.97	0.75	0.09	0.1	1.0
**rs10483727**	C	0.11	0.58	0.17	0.12	0.34	0.76	0.09	0.06	0.11	0.57	0.8	0.67
**rs1063192**	C	0.24	0.94	0.25	0.97	0.28	0.36	0.26	0.75	0.22	0.10	0.86	0.63
**rs9607469**	A	0.38	0.61	0.5	0.67	0.19	0.76	0.29	0.61	0.82	0.64	0.97	0.80
**rs1192415**	G	0.91	0.18	0.21	0.97	0.82	0.69	0.70	0.97	0.81	**0.021** ^Ω^ **(−10.9 ±4.7**)	**0.027** ^Ω^ **(0.05±0.02**)	0.87

^Ω^ = *P(*β± SE)

β = standard regression coefficient; SE = standard error; AL-axial length; CCT- Central corneal thickness, VCDR-vertical cup to disc ratio, IOP- intra ocular pressure.

*Obtained from linear regression, adjusted for age and gender

Bonferroni adjusted significant level< = 0.008 (0.05/6). Variation in no of samples depends on the availability of data.

### Genetic Association of the SNPs with IOP, CCT, AL and VCDR in POAG and controls

The analysis of endophenotypes (IOP, CCT, AL and VCDR) for potential association with the 6 SNPs was performed in POAG (HTG/NTG/combined) and controls using SPSS v17 (Table 4) after adjustment for age and gender; IOP was adjusted as an additional covariate for CCT and vice versa. The minor allele ‘C’ of rs1063192 (*CDKN2B*) showed association with decreased AL (additive model) in controls (*P* = 0.012 (β±SE = -0.388±0.15)). The minor allele ‘C’ of rs10483727 (*SIX1/SIX6)* was associated with decreased VCDR in combined cases and NTG (recessive model) (*P* = 0.014, β±SE = -0.062±0.025, *P* = 0.017, β±SE = -0.102±0.04 respectively). The minor allele ‘A’ of rs9607469 (*CARD10)* was associated with decreased VCDR in HTG (recessive model) (*P* = 0.022, β±SE = -0.141±0.06). The minor allele ‘T’ of rs1900004 (*ATOH7)* was associated with decreased VCDR in controls (additive; *P* = 0.013, β±SE = -0.028±0.02) and decreased IOP (additive; *P* = 0.042, β±SE = -0.042±0.02). However none of these significant *P* values passed Bonferroni correction.

**Table 4:** Association of the SNPs with CCT, VCDR, IOP, AL in controls and POAG (HTG/NTG/combined).

SNP	Minor allele	Control (n = 371)			NTG (n = 31)			HTG (n = 66)			Combined (n = 97)		
		β	SE	*P* *	β	SE	*P* *	β	SE	*P* *	β	SE	*P* *
**CCT**				N = 369			N = 27			N = 63			N = 87
**rs1900004**	T			0.34∞			0.46∞			0.31¶			0.29£
**rs3858145**	G			0.16¶			0.48£			0.21£			0.32∞
**rs10483727**	C			0.21∞			0.59£			0.08£			0.15¶
**rs1063192**	C			0.17∞			0.11£			0.08∞			0.27∞
**rs9607469**	A			0.4£			0.79¶			0.53£			0.27£
**rs1192415**	G			0.11 ∞			0.65£			0.38∞			0.14∞
**VCDR**				N = 363			N = 21			N = 45			N = 66
**rs1900004**	T	**−0.028**	**0.012**	**0.013¶**			.39∞			0.41¶			0.48¶
**rs3858145**	G			0.18∞			.42£			0.39£			0.43¶
**rs10483727**	C			0.27£	**−0.102**	**0.04**	**0.017∞**			0.3∞	**−0.062**	**0.025**	**0.014∞**
**rs1063192**	C			0.19¶			0.13£			0.18£			0.52£
**rs9607469**	A			0.27£			0.4£	**−0.141**	**0.06**	**0.022∞**			0.07∞
**rs1192415**	G			0.12∞			0.16¶			0.52£			0.34¶
**IOP**				N = 369			N = 27			N = 54			N = 81
**rs1900004**	T			0.2£			0.23¶	**−0.042**	**0.02**	**0.042¶**			0.29£
**rs3858145**	G			0.62∞			0.21¶			0.2£			0.27∞
**rs10483727**	C			0.11£			0.23∞			0.07∞			0.75∞
**rs1063192**	C			0.14£			0.72£			0.59∞			0.36£
**rs9607469**	A			0.69£			0.57£			0.21£			0.68∞
**rs1192415**	G			0.21¶			0.15∞			0.51£			0.59£
**AL**				**N = 74**									**N = 39**
**rs1900004**	T			0.49∞			-			-			0.11∞
**rs3858145**	G			0.29£			-			-			0.45£
**rs10483727**	C			0.26∞			-			-			0.21∞
**rs1063192**	C	**−0.388**	**0.15**	**0.012¶**			-			-			0.17£
**rs9607469**	A			0.22£			-			-			0.46∞
**rs1192415**	G			0.62£			-			-			0. 07£

β = standard regression coefficient

SE = standard error

£- dominant model

∞- recessive model

¶-additive model

*Obtained from linear regression

Bonferroni adjusted significant level< = 0.008 (0.05/6)

### Gene-gene interaction analysis

Since in POAG, interaction of several genes has been proposed, we carried out logistic regression analysis for studying the interaction between the 6 SNPs (coded as in Table 5) [37]. Significant interaction was observed in dominant model for the explanatory variable 2 (model) in HTG cases between rs10483727-rs1063192 (*SIX6-CDKN2B*) (Table 6) (“CC/CT”-“TT” (*P* = 0.04, OR = 2.5, 95%CI: 1.0–6.1)) and rs10483727-rs9607469 (*SIX6-CARD10*) (“CC/CT”-“GG” (*P* = 0.031,OR = 3.1(1.1–8.6)).

In recessive model, we observed significant interactions (NTG group) between rs3858145 with rs10483727 (*ATOH7-SIX6*) (“GG”-“TT/CT”, (*P* = 0.037, (OR = 2.6(1.06–6.5) explanatory variable 1).

Additive model replicated the interactions between (i) *SIX6-CDKN2B* and *SIX6-CARD10* showed in HTG group and (ii) *ATOH7-SIX6* in NTG group (S1 Table). In addition to this, we also observed significant interactions between *ATOH7-CDC7* and *SIX6-CDC7* among the HTG group (“CC”-“GG” (*P* = 0.014,OR = 4.3(1.3–13.6)) and “CC”- “GG” (*P* = 0.042,OR = 6.4(1.1–37.9)) (S1 Table). No significant interactions were observed between *ATOH7-CDKN2B*, *ATOH7-CARD10*, *SIX6-CDC7*, *CDKN2B-CARD10*, *CDKN2B-CDC7* and *CARD10-CDC7*.

**Table 5:** Coding of genotypes in dominant and recessive model analysis of interaction between genes.

SNPs rs10483727/ rs1063192/ rs9607469/ rs1192415	Genotype code*	SNPs in *ATOH7* (rs1900004/rs3858145)	
		MM and MW	WW
**Dominant model**	**MM and MW**	3	1
	**WW**	2	0
		**MM**	**WW and MW**
**Recessive model**	**MM**	3	2
	**WW and MW**	1	0

* MM—mutant homozygote, MW- heterozygote, WW- wild homozygote

rs1900004: MM- TT, MW-CT, WW-CC; rs3858145 and rs1192415: MM- GG, MW-GA, WW-AA; rs10483727 and rs1063192: MM- CC, MW-CT, WW-TT; rs9607469: MM- AA, MW-GA, WW-GG

**Table 6:** Analysis for the interaction between SNPs in HTG/NTG by dominant and recessive models.

	**Dominant model**						
		**HTG**			**NTG**		
**SNP1**	**SNP2**	**1**	**2**	**3**	**1**	**2**	**3**
rs1900004	rs10483727	0.1	0.9	0.2	0.6	0.9	0.3
	rs1063192	0.1	0.1	0.7	0.7	0.4	0.3
	rs9607469	0.8	0.9	0.7	0.7	0.3	0.6
	rs1192415	0.1	0.3	0.4	0.3	0.9	0.4
rs3858145	rs10483727	0.4	0.7	0.3	0.8	0.3	0.2
	rs1063192	0.2	0.2	0.8	0.9	0.2	0.2
	rs9607469	0.4	0.6	0.6	0.8	0.2	0.4
	rs1192415	0.2	0.6	0.3	0.9	0.3	0.2
rs10483727	rs1063192	0.2	0.04 (2.5(1.0–6.1)) *	0.1	0.7	0.8	0.7
	rs9607469	0.3	0.031 (3.1(1.1–8.6)) *	0.1	0.8	0.5	0.9
	rs1192415	0.8	0.4	0.1	0.1	0.4	0.9
rs1063192	rs9607469	0.4	0.6	0.9	0.1	0.3	0.7
	rs1192415	0.8	0.2	0.4	0.4	0.3	0.9
rs9607469	rs1192415	0.9	0.2	0.7	0.4	0.2	0.6
	**Recessive model**						
**SNP1**	**SNP2**	**1**	**2**	**3**	**1**	**2**	**3**
rs1900004	rs10483727	0.5	0.6	1.0	0.2	0.1	0.9
	rs1063192	0.9	0.7	0.4	0.8	0.5	0.1
	rs9607469	0.6	0.7	1.0	0.3	0.8	0.9
	rs1192415	0.6	0.1	1.0	0.2	0.1	0.9
rs3858145	rs10483727	0.6	0.6	0.7	0.037 (2.6(1.059–6.497)) *	0.09	0.5
	rs1063192	0.7	0.5	0.9	0.1	0.7	0.4
	rs9607469	0.8	1.0	1.0	0.1	0.5	0.5
	rs1192415	1.0	0.3	0.1	0.1	0.3	0.2
rs10483727	rs1063192	0.5	0.2	0.9	0.1	0.6	0.9
	rs9607469	0.5	0.4	0.9	0.1	0.9	0.9
	rs1192415	0.7	0.5	0.1	0.2	0.3	0.3
rs1063192	rs9607469	0.4	0.7	0.9	0.9	0.7	0.9
	rs1192415	0.6	0.2	0.4	0.8	0.2	0.9
rs9607469	rs1192415	0.9	0.2	0.6	0.9	0.2	0.9

* *P* value (OR(95%CI))

## Discussion

POAG is a complex genetic disorder exhibiting clinical and genetic heterogeneity. Identification of the genetic variants associated with the endophenotypes has been proven as one of the effective method, in disorders with complex inheritance pattern [13]. Literature reveals significant association of these genes with POAG in several populations like US Caucasians [38], Japanese [23], Rotterdam study cohort I and II [22], Australian and UK twin cohort [20], Afro-Caribbean [39] etc. In contrast, our study did not show significant association for any of these SNPs with POAG which could be possibly attributed to difference in the population. A similar observation has been earlier reported in East Indian cohort [34]. The sample size of the current study is another factor that reduces the power to detect the true associations.

We analyzed for the potential association of SNPs near *ATOH7*, *CDKN2B*, *SIX6*, *CARD10*, and *CDC7* with AL, CCT, IOP and VCDR. The other optic disc parameters like ODA, were not included for the current study due to the unavailability of data for these samples.

*CDKN2B* (p15), a cyclin dependant kinase inhibitor type 2B gene, acts as a tumour suppressor in the retinoblastoma pathway. This gene along with the adjacent genes like *CDKN2A*, *CDKN2B-AS* is involved in cell signalling pathways and has been associated with POAG [40]. The ‘C’ allele of rs1063192 was previously associated with a decreased risk for POAG in Afro-Caribbean population [39] and with decreased VCDR [22,38], while the other allele “T” has been associated with increased VCDR and NTG [23]. We did not observe any significant association with POAG/VCDR. A similar observation was made by Vishal et al in East Indian population, thus suggesting that rs1063192 is not associated with VCDR in Indian population [34]. The ‘C’ allele of rs1063192 was correlated with decreased AL in controls; which indirectly suggests less risk for POAG in the study population. This is the first report on the association of *CDKN2B* with AL and needs to be replicated for further disease correlations.

*ATOH7* is a transcription factor, required for the genesis of retinal ganglion cells [41,42]. The ectopic expression of *ATOH7* was shown to increase the number of differentiated retinal ganglion cells in *invitro* models [43,44]. Mutations in this gene have been associated with autosomal recessive persistent hyperplasia and optic nerve hypoplasia [20,45]. Earlier GWAS analysis has shown association of SNPs rs1900004 [22], rs3858145 [20] and rs7916697 with ODA and VCDR [21] in various cohorts. In the present study we have analysed 2 SNPs (rs1900004 and rs3858145) in which we were able to replicate the association of rs1900004 with decreased VCDR in whole cohort (additive and recessive models) as well as controls (additive models). Further the ‘T” allele of rs1900004 also showed novel associations with decreased i) AL (whole cohort- recessive model) and ii) IOP (HTG- additive model). The ‘A’ allele of rs3858145 had been previously associated with increased cup and disc area in Australian and UK cohort [20], but in the current study we did not find any significant association.

*SIX1*/*SIX6* complex belong to the homeoprotein group of proteins involved in development. SNP rs10483727 (T-allele) has earlier been associated with increased VCDR [22,23,38,46], IOP [28,46] and POAG in several GWAS studies [21,22,47,48,49]. In the current study, the ‘C’ allele was observed as minor allele in contrast to that observed in other populations and it was associated with decreased VCDR in NTG and combined cases.

*CDC7* a cell division cycle protein is critical for G1/S transition. Earlier studies have shown association of *CDC7* with increased ODA and POAG in various populations [21,22,39]. Interestingly, in the present study we have observed an indirect risk for POAG, as association was observed with subjects having thin CCT and increased VCDR, major risk factors for POAG. This novel association of *CDC7* with CCT has to be replicated for further disease correlation.

*CARD10* belongs to caspase recruitment domain family member 10 which is involved in regulation of caspase activation and apoptosis (via NF-kappaB) in addition to its role in assembling membrane associated signalling complexes. SNP rs9607469 (A-allele) in this gene has been associated with increased ODA [21] by GWAS. A recent meta analysis for VCDR identified SNP rs5756813 near *CARD10* as one of the loci with positive correlation for VCDR [50]. In the current study we have also replicated the association of *CARD10* with VCDR wherein the ‘A’ allele of rs9607469 is associated with decreased VCDR. We did not identify any association with POAG as seen in other reports.

Analysis of potential interaction between the SNPs was carried out using logistic regression analysis. We were able to replicate the association between the genes *ATOH7- SIX6* among NTG as shown by Fan etal [38] and also between genes *SIX6-CDKN2B* among HTG as shown by Iglesias et al [46]. Novel interactions, that were statistically significant was observed between *SIX6-CARD10*, *SIX6-CDC7* and *ATOH7-CDC7* among HTG patients. Chen et al showed that though individually the SNPs were not associated with POAG, an introduction of interaction term brought significant result [51]. In our study we also did not observe any significant association with POAG but when analysed with interaction effects, the association was ascertained thus substantiating the fact that complex disorders such as glaucoma, result from interactions between several genes. These interactions among genes like *ATOH7*, *SIX6*, *CDKN2B* and *CDC7* suggests a putative additive role of the developmental and growth signalling pathways in POAG as hypothesized in many studies [46,48].

In summary, our results suggest that a probable interaction among the candidate genes for QTs play a major role in determining the individual’s susceptibility to POAG, similar to other complex diseases. The current study reports a potential interaction between SNPs near *ATOH7—CDC7*, *SIX6-CARD10*, *SIX6-CDC7* genes with POAG for the first time in the literature. SNPs near the genes (*ATOH7*, *SIX6*, *CDKN2B*, *CARD10*, and *CDC7*) that did not show individual association with the disease however correlated with increased risk when analysed for interactions. We also replicated the association of these genes with QTs as observed in other population. Additionally, we report here a novel association of SNPs near (i) *ATOH7* and *CDKN2B* with AL (ii) *CDC7* with CCT and iii) *ATOH7* with IOP. However as none of these *p*-values (linear regression analysis) survived Bonferroni correction which is a stringent criterion to prevent type one errors, replication and functional studies are necessary for further conclusions.

## Supporting Information

S1 Table Analysis of interaction between SNPs in HTG/NTG by additive model. (a) Coding of genotypes (b) Results from logistic regression analysis.
(DOCX)
