# Supplementary material for: Genetic Association of SNPs near ATOH7, CARD10, CDKN2B, CDC7 and SIX1/SIX6 with the Endophenotypes of Primary Open Angle Glaucoma in Indian Population
Source: PLoS One. 2015 Mar 23;10(3):e0119703. doi: 10.1371/journal.pone.0119703 (PMC4370747; doi:10.1371/journal.pone.0119703)
Supplement: S1 Table — (a) Coding of genotypes (b) Results from logistic regression analysis. (DOCX) [file pone.0119703.s001.docx]

**S1 Table. Analysis of interaction between SNPs in HTG/NTG by additive model.** (a) Coding of genotypes (b) Results from logistic regression analysis

| 1. **Coding of genotypes for additive model** | | | | | | | | | | | | | | | |
| --- | --- | --- | --- | --- | --- | --- | --- | --- | --- | --- | --- | --- | --- | --- | --- |
| 0 | 1 | | 2 | 3 | | | 4 | 5 | | 6 | | 7 | | 8 | |
| ww/ww | mm/mm | | mm/wm | mm/ww | | | wm/mm | wm/wm | | wm/ww | | ww/mm | | ww/wm | |
| 1. **Logistic regression results for Additive model** | | | | | | | | | | | | | | | |
| **HTG** | | | | | | | | | | | | | | | |
|  | **1** | **2** | | | **3** | **4** | | | **5** | | **6** | | **7** | | **8** |
| rs1900004-rs10483727 | 1 | 0.12 | | | 0.76 | 0.44 | | | 0.34 | | 0.99 | | 0.4 | | 0.23 |
| rs1900004-rs1063192 | 0.54 | 0.45 | | | 0.89 | 0.81 | | | 0.63 | | 0.17 | | 0.7 | | 0.15 |
| rs1900004-rs9607469 | 1 | 0.76 | | | 0.51 | 0.89 | | | 0.91 | | 0.72 | | 0.76 | | 0.77 |
| rs1900004-rs1192415 | 1 | 0.16 | | | 0.85 | 0.4 | | | 0.48 | | 0.24 | | **0.014(4.3(1.3-13.6)) ^*^** | | 0.18 |
| rs3858145-rs10483727 | 0.76 | 0.24 | | | 0.65 | 0.91 | | | 0.38 | | 0.62 | | 0.24 | | 0.72 |
| rs3858145-rs1063192 | 0.88 | 0.21 | | | 0.86 | 0.22 | | | 0.91 | | 0.13 | | 1 | | 0.43 |
| rs3858145-rs9607469 | 0.83 | 0.87 | | | 0.88 | 0.69 | | | 0.84 | | 0.53 | | 0.99 | | 0.69 |
| rs3858145-rs1192415 | 0.09 | 0.27 | | | 0.79 | 0.25 | | | 0.65 | | 0.44 | | 0.22 | | 0.26 |
| rs10483727-rs1063192 | 1 | 0.24 | | | 0.14 | **0.039(4.2(1.1-16.1)) ^*^** | | | 0.26 | | **0.04(2.6(1.04-6.5)) ^*^** | | 0.43 | | 0.24 |
| rs10483727-rs9607469 | 1 | 0.16 | | | 0.12 | **0.041(4.5(1.07-19.2)) ^*^** | | | 0.19 | | **0.031(3.2(1.1-8.9)) ^*^** | | 0.83 | | 0.15 |
| rs10483727-rs1192415 | **0.042(6.4(1.07-37.9))^*^** | 0.23 | | | 0.65 | 0.4 | | | 0.1 | | 0.22 | | 0.09 | | 0.92 |
| rs1063192-rs9607469 | 1 | 0.96 | | | 0.38 | 0.36 | | | 0.54 | | 0.37 | | 0.5 | | 0.29 |
| rs1063192-rs1192415 | 0.47 | 0.4 | | | 0.58 | 0.22 | | | 0.69 | | 0.22 | | 0.54 | | 0.72 |
| rs9607469-rs1192415 | 0.71 | 0.63 | | | 0.37 | 0.08 | | | 0.86 | | 0.3 | | 0.8 | | 0.96 |
| **NTG** | | | | | | | | | | | | | | | |
| rs1900004-rs10483727 | 1 | 0.06 | | | 1 | **0.001(0.1(.025-.415)) ^*^** | | | 0.72 | | 0.64 | | 1 | | 0.81 |
| rs1900004-rs1063192 | 0.07 | 0.21 | | | 0.76 | 1 | | | 0.32 | | 0.39 | | 0.88 | | 0.31 |
| rs1900004-rs9607469 | 1 | 0.47 | | | 0.33 | 0.37 | | | 0.84 | | 0.28 | | 1 | | 0.63 |
| rs1900004-rs1192415 | 1 | 0.18 | | | 0.9 | 0.18 | | | 0.92 | | 0.8 | | 0.82 | | 0.14 |
| rs3858145-rs10483727 | 0.2 | **0.042(0.1(0.012-.92)) ^*^** | | | 0.23 | **0.022(0.08(0.009-0.69)) ^*^** | | | 0.54 | | 0.19 | | 1 | | 0.31 |
| rs3858145-rs1063192 | 0.24 | 0.14 | | | 0.12 | 1 | | | 0.29 | | 0.35 | | 0.54 | | 0.48 |
| rs3858145-rs9607469 | 0.34 | 0.58 | | | 0.06 | 0.82 | | | 0.61 | | 0.37 | | 1 | | 0.67 |
| rs3858145-rs1192415 | 0.14 | 0.35 | | | 0.08 | 0.19 | | | 0.57 | | 0.51 | | 1 | | 0.74 |
| rs10483727-rs1063192 | 1 | 0.25 | | | 0.38 | 1 | | | 0.9 | | 0.81 | | 0.54 | | 0.26 |
| rs10483727-rs9607469 | 1 | 0.68 | | | 0.14 | 0.94 | | | 0.76 | | 0.86 | | 0.88 | | 0.45 |
| rs10483727-rs1192415 | 0.56 | 0.17 | | | 0.29 | 0.7 | | | 0.19 | | 0.52 | | 1 | | 0.11 |
| rs1063192-rs9607469 | 1 | 0.65 | | | 0.94 | 0.76 | | | 0.74 | | 0.27 | | 0.5 | | 0.09 |
| rs1063192-rs1192415 | 1 | 0.44 | | | 1 | 0.27 | | | 0.36 | | 0.45 | | 0.73 | | 0.26 |
| rs9607469-rs1192415 | 1 | 1 | | | 0.99 | 0.78 | | | 0.34 | | 0.1 | | 0.98 | | 0.35 |

**^*^ *P* value (OR(95%CI))**
